# OLDER ADULTS’ SAVORING ABILITY IS ASSOCIATED WITH LONGITUDINAL CHANGE IN WELL-BEING

**DOI:** 10.1093/geroni/igad104.3505

**Published:** 2023-12-21

**Authors:** Jacquelyn Stephens, Laurel Mertz, Jennifer Smith

**Affiliations:** Mather Institute, Evanston, Illinois, United States; Mather Institute, Evanston, Illinois, United States; Mather Institute, Evanston, Illinois, United States

## Abstract

Well-being in older adults is an important concern as the aging population grows, as is identifying modifiable factors that contribute to well-being over time. The ability to savor the moment (i.e., to notice and enhance one’s emotional response to positive events) has been shown to be associated with greater well-being, though few studies have examined these relationships longitudinally or in older adults. The current study examined whether savoring ability moderated the trajectory of older adults’ life satisfaction and depressive symptoms over time. A sample of 5777 older adults (Mage = 83.83, SD = 6.61; 66.6% female), recruited from Life Plan Communities, completed four consecutive annual surveys. Multilevel growth curve models revealed main effects of small but significant declines in well-being over time (life satisfaction: B = -.09, SE = .01, p < .001; depressive symptoms: B = .13, SE = .01, p < .001), after accounting for age, gender, education, life stress, and health. Interactions between time and savoring ability indicated that participants with greater savoring ability were better able to maintain their levels of well-being (i.e., smaller decline in life satisfaction [B = .05, SE = .01, p < .01] and smaller increase in depressive symptoms [B = -.10, SE = .01, p < .01]). In contrast, individuals lower in savoring ability experienced less favorable well-being trajectories. Savoring is a malleable emotion regulation ability that is associated with attenuated declines in well-being over time. Implications for savoring interventions in older adults will be discussed.

